# Impact of Incorporating Future Mandatory Price Reductions with Generic Drug Entry on the Cost-Effectiveness of New Drugs: A Policy Simulation Study of Dupilumab in Atopic Dermatitis Treatment

**DOI:** 10.3390/healthcare12090938

**Published:** 2024-05-02

**Authors:** Maryanne Kim, Guiguan Quan, Youran Noh, Song Hee Hong

**Affiliations:** 1 College of Pharmacy, Seoul National University, Seoul 08826, Republic of Korea; 2 Research Institute of Pharmaceutical Sciences, Seoul National University, Seoul 08826, Republic of Korea

**Keywords:** drug life-cycle pricing, drug benefit policy, economic evaluation, policy simulation study, health technology assessment, generic entry, dupilumab, atopic dermatitis

## Abstract

The introduction of high-cost medications often poses challenges in achieving cost-effectiveness for drug insurance coverage. Incorporating future price reductions for these medications may enhance their cost-effectiveness. We examined the influence of future cost reductions mandated by the national insurer’s equal pricing for equivalent drugs (EPED) policy on the cost-effectiveness of dupilumab, a biologic drug for moderate to severe atopic dermatitis in the Korean healthcare system. We conducted a policy simulation study using semi-Markovian cost utility analysis of dupilumab in combination with supportive care (SC) versus SC alone, with and without the EPED policy adjustment. The EPED would lower dupilumab’s price to 70% following the entry of a biosimilar drug in 10.3 years. Scenario analyses quantified the impact of changing time to the EPED, chemical versus biological designation, response criteria, discount rates, and time horizons on the Incremental Cost-Effectiveness Ratio (ICER) and acceptability with and without EPED adjustment. The EPED adjustment of dupilumab’s future price significantly improved its cost-effectiveness, with a 9.7% decrease in ICER and a substantial 14.6% increase in acceptability. Assuming EPED in 5 years, the ICER fell below the predefined willingness-to-pay threshold. If dupilumab were a chemical drug, EPED adjustment demonstrated a 19.1% increase in acceptability. Incorporating future cost reductions via the EPED system in economic evaluations is crucial, especially for drugs facing imminent generic entry. This study underscores the importance of EPED adjustment in the cost-effectiveness analysis of innovative medications, especially for those nearing willingness-to-pay thresholds.

## 1. Introduction

The introduction of highly expensive new drugs to the market often creates challenges in meeting the incremental cost-effectiveness ratio (ICER) threshold for drug insurance coverage [1,2,3,4]. These challenges become even more complicated when future drug price changes are not factored in to economic evaluation. A study in the UK reports that future price reductions for new drugs associated with their generic drug entry are, on average, equivalent to an annual reduction of 3.8% per year over the drug’s lifespan [5]. Factoring in this price reduction for economic evaluation leads to an improvement of 24% to 46% in cost-effectiveness [6]. Considering the expansion of the patient population who uses the drug over time, the impact the future price reduction has on cost-effectiveness cannot be overlooked [7,8]. Moreover, it raises questions of neutrality when generic costs are used for the comparator but not for the new drug [9,10,11,12,13].

Accounting for future price reductions for the new drug is not an easy task because of the difficulty in predicting the price reductions and the proportion of patients who will switch from the new original drug to its generic versions [9,14]. However, the situation is different in Korea due to its national drug benefit policy, which unilaterally lowers the drug cost upon the entry of the first generic version based on the principle of “equal pricing for equivalent drugs” (EPED) [15,16,17,18,19]. The EPED policy is implemented differently for chemical and biologic drugs. For chemical drugs, it follows a step-wise reduction strategy with initial differential pricing between the original and the first generic (70% for the original and 59.5% for the first generic), followed by subsequent equal pricing (53.5%) a year after the entry of the first generic. In contrast, for biologic drugs, the EPED takes a simpler form with an immediate 30% reduction upon the entry of the first biosimilar drug. After implementation of EPED, drug prices rarely experience significant price competition, remaining close to the ceiling price allowed by the EPED with limited variance [16,18,20,21]. Consequently, the EPED effectively eliminates uncertainty in estimating drug costs post-entry for economic evaluations.

The primary aim of this study was to investigate how cost reductions resulting from EPED policy impact the cost-effectiveness of a new drug, particularly in comparison to a low-cost generic comparator. The substantial price difference between the new drug and the generic comparator poses a challenge for the new drug to demonstrate cost-effectiveness, as it must show a significant improvement in effectiveness to justify its higher cost [22,23]. In contrast, comparing the new drug to an expensive brand comparator may only require a minor improvement due to the minimal price gap. In cases involving a generic comparator, the impact of EPED-induced price reductions on cost-effectiveness would be more pronounced because the reduction only applies to the new drug with the higher price. To the contrary, when comparing to a brand comparator, the impact would be negligible not only due to minimal price differences but also because both alternatives undergo price reductions. Furthermore, these price reductions could even negatively affect the cost-effectiveness when the brand comparator undergoes price reduction sooner than the new drug.

As a representative example of the investigation, we selected dupilumab, a biologic drug indicated for moderate to severe atopic dermatitis (AD). The drug was chosen based on the following criteria: (1) the drug is chronically used for a lifetime treatment, (2) the comparator is supportive care (SC) consisting of inexpensive treatments such as generic materials, and (3) the economic evaluation of the drug without factoring in the future cost reduction exceeds the cost-effectiveness threshold set by Korea’s national health insurance program. Dupilumab, approved for managing moderate to severe atopic dermatitis in 2018, satisfies all the criteria. However, it was denied drug formulary listing for national health insurance (NHI), likely due to its cost-effectiveness compared to SC exceeding the NHI’s willingness-to-pay (WTP) threshold. Nevertheless, it managed to enter a risk-sharing agreement (RSA), wherein a percentage of the drug expenditure exceeding a specified threshold is clawed back for the NHI [24,25]. The case of dupilumab serves as a compelling example where the incorporation of EPED-induced price reduction could have potentially altered the outcome of the economic evaluation. 

Additionally, this study aimed to analyze the impact of EPED adjustment on the value-based price of dupilumab at different WTP thresholds. Lastly, this study aimed to quantify the extent to which different EPED configurations (time to EPED and biosimilar/chemical-based EPED), time horizon, treatment response criteria, and discount rate on the EPED-adjusted cost-effectiveness.

The findings from this research will inform Health Technology Assessment (HTA) agencies of the significance of incorporating “future drug price changes or drug life-cycle pricing” in the economic evaluation of new original drugs. By considering the dynamic changes in drug costs over time, HTA agencies can make more informed decisions on drug formulary listings.

## 2. Materials and Methods

### 2.1. Study Design

We conducted a semi-Markovian cost-utility analysis comparing dupilumab plus SC versus SC only, with and without factoring in EPED, from the perspective of the healthcare system in Korea. The perspective was recommended in the “Guidelines for Economic Evaluation of Pharmaceuticals” published by the Health Insurance Review and Assessment Service (HIRA) in Korea [26]. Following the guidelines, we excluded non-direct healthcare costs as well as indirect costs. This approach minimizes the uncertainty of costs incurred outside the healthcare system.

We compared costs and quality-adjusted life years (QALYs) between patients in each therapy for treatment of moderate to severe AD, with or without adjusting for EPED. Dupilumab therapy consisted of a 300 mg dose every two weeks after a 600 mg loading dose, in combination with SC, where SC consisted of emollients. Our findings were reported following the guidelines of the Consolidated Health Economic Evaluation Reporting Standards, which are provided in the Supplementary Table S1 [27].

### 2.2. Model Structure

The decision tree linked to a state-transition semi-Markov model was built based on two existing cost-effectiveness analyses conducted for dupilumab [28,29]. The model depicted health state transitions for a cohort of moderate to severe Korean AD patients over a lifetime horizon. The model operated on 4-month cycles, and costs and QALYs were discounted at an annual rate of 4.5%, following the Korean economic evaluation guideline [26].

**Figure 1:**
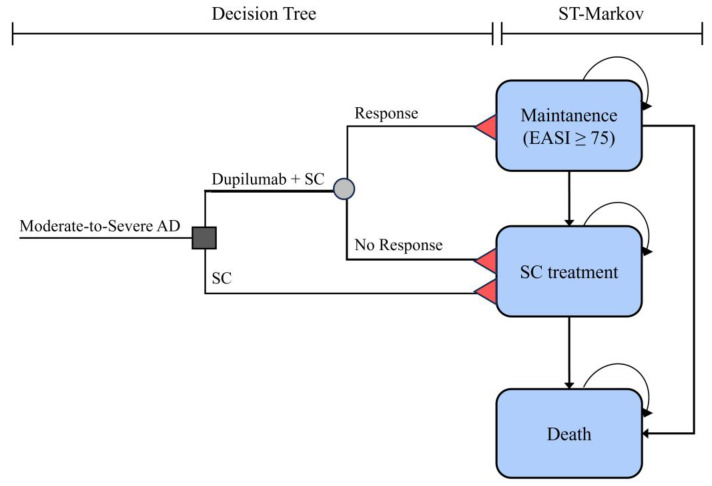
Transition model structure. Black arrows indicate transitions between health states. Red triangles indicate terminal states for each treatment arm. AD, atopic dermatitis; EASI, eczema area and severity index; SC, supportive care; ST, state-transition. Color has no significant meaning.

Moderate to severe AD patients were assigned to either the dupilumab plus SC group or the SC group during the 4-month decision tree. Among the dupilumab plus SC group, those who achieved at least a 75% decrease in the Eczema Area and Severity Index (EASI-75) entered the state of “dupilumab maintenance” (or response) in the Markov model, while those who did not achieve the improvement or who discontinued dupilumab entered the state of “SC treatment” (or no response) (Figure 1). Patients in the SC group were assigned to the state of “SC treatment” after 4 months. Patients in the response state who were receiving dupilumab maintenance therapy could either continue to respond, make a transition to the “SC treatment” state, or face the possibility of death. However, “SC treatment” could not transition back to the “dupilumab maintenance” state. We assumed that the likelihood of patient mortality was influenced by age but not by their chosen therapy options or the severity of AD.

### 2.3. Patients

The patient cohort had an average age of 38 years, with 54% men, which closely resembled the population in the two randomized, placebo-controlled, phase 3 trials (SOLO 1: NCT02277743; SOLO 2: NCT02277769) [30]. We assumed that the cohort consisted of 48% severe AD and 52% moderate AD patients who were not responsive to tropical therapy or for whom topical therapies were not medically advised. Patient mortality rates were derived from the Korean population life tables [31].

### 2.4. Transition Probabilities

The transition probability to the response state from the initial state for each therapy was estimated based on the SOLO trials (Table 1) [30]. The probability of achieving at least a 75% decrease in EASI was 47.7% for dupilumab q2w (every two weeks) and 13.3% for SC patients at week 16. An annual discontinuation rate of 6.3% was observed for dupilumab, representing those who initially responded transitioning back to a no response state upon discontinuing the treatment [29]. The relapse rate for SC was 65.8% at week 16 [32]. All the probabilities were adjusted to fit a 4-month probability to align with the cycle length [33].

### 2.5. Adverse Events

Three adverse events (injection site reaction, allergic conjunctivitis, and infectious conjunctivitis) significantly affect the estimation of dupilumab treatment costs. The occurrence rates of these adverse events were derived from the SOLO trials (Table 1) [30]. It was assumed that injection site reactions would only occur once for dupilumab. On the other hand, both allergic and infectious conjunctivitis were assumed to occur in each and every subsequent cycle.

### 2.6. Cost Estimation

The maximum allowable cost for a 300 mg injection of dupilumab in Korea was KRW 710,000, equivalent to USD 620.3 as of 2021. The annual cost of dupilumab therapy, administered every two weeks, was approximately KRW 18,460,000 (USD 16,128). For SC, due to its low cost and the challenges in accurately estimating the expenses associated with emollient use, a simplified decision was made to omit the cost of emollients from the model [28,29].

Other healthcare costs associated with various medical services, such as physician visits, medical tests, procedures, and emergency care, were obtained from a study that examined the economic burden of Korean AD patients based on severity [35]. In the context of the analysis, we assumed that responders would have healthcare costs similar to those of patients with mild AD severity. Conversely, individuals who did not respond were assumed to have healthcare costs similar to the mean costs of moderate and severe AD patients. We assumed that the healthcare costs of each state do not vary depending on the interventions. 

The annual treatment costs of adverse events were obtained from the 2021 medical statistics provided by the HIRA (Supplementary Table S2) [36]. This data source offers details about the patient count, days of visits, number of claims, total medical expenses, and insurance co-payments for each Korean Standard Classification of Diseases (KSCD). By considering the number of claims, we calculated the weighted averages of medical expenses for each adverse event identified by KSCD codes. All costs from previous years were adjusted for inflation and presented in terms of 2021 KRW. 

### 2.7. QALY Estimation

The health-related quality of life (HRQoL) for each health state was determined using 5-dimension 3-level EuroQol (EQ-5D) utilities obtained from the SOLO clinical trials [29,30,37]. The utility for the non-responded state was derived from the baseline score of 0.61 in the placebo group and 0.63 in the dupilumab q2w group. The utility of the responded state was calculated by adding the baseline utility score to the least squared mean changes. These changes were derived from patients who experienced improvements in their outcomes. We assumed that the utility of the baseline population has a variance different from that of the population that responded to the treatment. 

### 2.8. EPED Configuration

The EPED exerts different effects depending on when the EPED will occur and how much cost reduction the EPED will bring. The timing of the EPED, determined by factors such as patent expiration and generic drug approval, signifies when generic entry-induced price reductions take place. Upon the entry of a generic drug into the market, immediate reductions in drug prices occur. The magnitude of price reduction depends on whether the drug is designated as chemical or biological. For biological drugs like dupilumab, the EPED mandates a categorical 30% reduction, irrespective of whether it is the original drug or its follow-ons. On the other hand, for chemical drugs, the EPED follows a step-wise reduction strategy, starting with differential pricing between the original and the first generic (70% for the original and 59.5% for the first generic), followed by subsequent equal pricing (53.5%) a year after the entry of the first generic.

**Figure 2:**
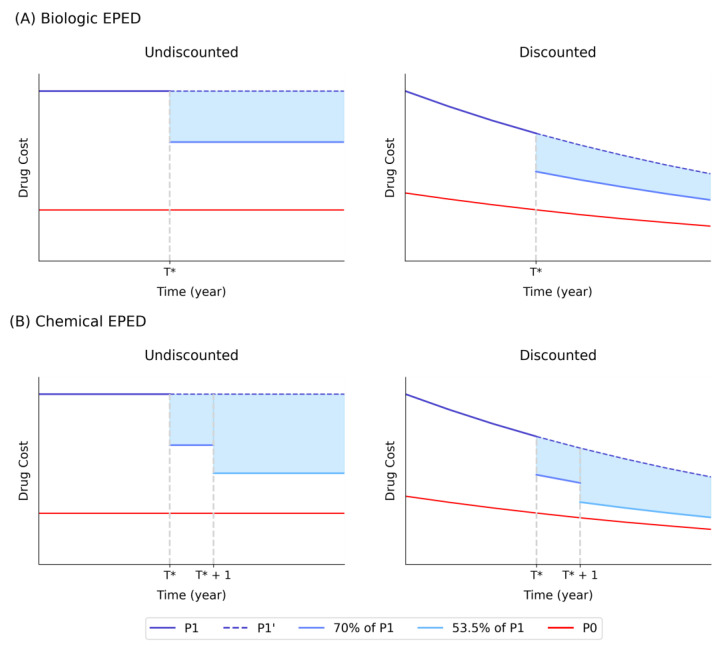
Drug cost change over the time for (**A**) biological drugs and (**B**) chemical drugs. The blue area represents the lifetime savings from EPED in new drug costs. P1, new drug cost (pre-entry); P1’, the previous cost extended; P0, comparator drug cost; T*, time of first generic entry.

The lifetime savings from EPED in new drug costs is described as the blue shaded area of Figure 2 below. This study used the EPED configuration, which implements a 30% cost reduction in year 10.3 to calculate the adjusted ICER. This configuration is used because dupilumab, as a large-molecule biological, is expected to face the first biosimilar in May 2031, which is approximately 10.3 years from January 2021, the year at which the application for NHI coverage should have been submitted [38]. The mathematical framework of the EPED policy is shown in the Supplementary Information S1.

### 2.9. Analysis 

The effect of the EPED price reduction on the cost-effectiveness of dupilumab plus SC versus SC was evaluated as a percentage improvement in the ICER as well as in acceptability before and after adjusting for the EPED price reduction. For the probabilistic sensitivity analysis (PSA), we assigned distributions to the model parameters, as shown in Table 1. Monte Carlo simulation techniques were then used to iteratively draw 1000 samples from these distributions, allowing the ICERs to be calculated for each iteration of the simulation. Acceptability was determined based on a graph depicting the probability that dupilumab is considered cost-effective for a range of maximum WTPs. From the graph, the acceptability of dupilumab was read at KRW 40,052,159/QALY (equivalent to USD 34,992/QALY), the conventionally recommended value of 1 GDP per capita in 2021, as there is no official WTP threshold in Korea [39,40]. We examined how the adjustment of the EPED policy affects the value-based acceptance price for the drug reimbursement across different WTP thresholds. The thresholds were determined as the median of all the ICERs accepted by the HIRA from 2014 to 2021 for different diseases: anticancer drugs (KRW 45,320,000/QALY), rare disease drugs (KRW 38,400,000/QALY), and general drugs (KRW 17,170,000/QALY) [41].

Scenario analyses were then performed to examine the potential impact of different EPED configurations on the estimation of the ICER and the acceptability. We explored variations in time to the EPED at 5, 7, and 12 years. To evaluate the influence of step-wise EPED strategies for chemical drugs, we changed the price reductions sequentially to measure the change in the ICER. Other scenario analyses included using the EASI-50 threshold for response criteria [30], a discount rate of 3.0%, and shorter time horizons of 30 years and 20 years.

A one-way sensitivity analysis was performed to assess the impact of uncertainty in the model parameters on the outcomes of the EPED adjustment, specifically the change in the ICER before and after the adjustment. This analysis involved varying each parameter over its 95% confidence intervals while keeping all other parameters constant.

We used Microsoft Excel and Python 3.8 for performing and analyzing the study.

## 3. Results

### 3.1. Cost-Effectiveness of Dupilumab after EPED Policy Adjustment

**Figure 3:**
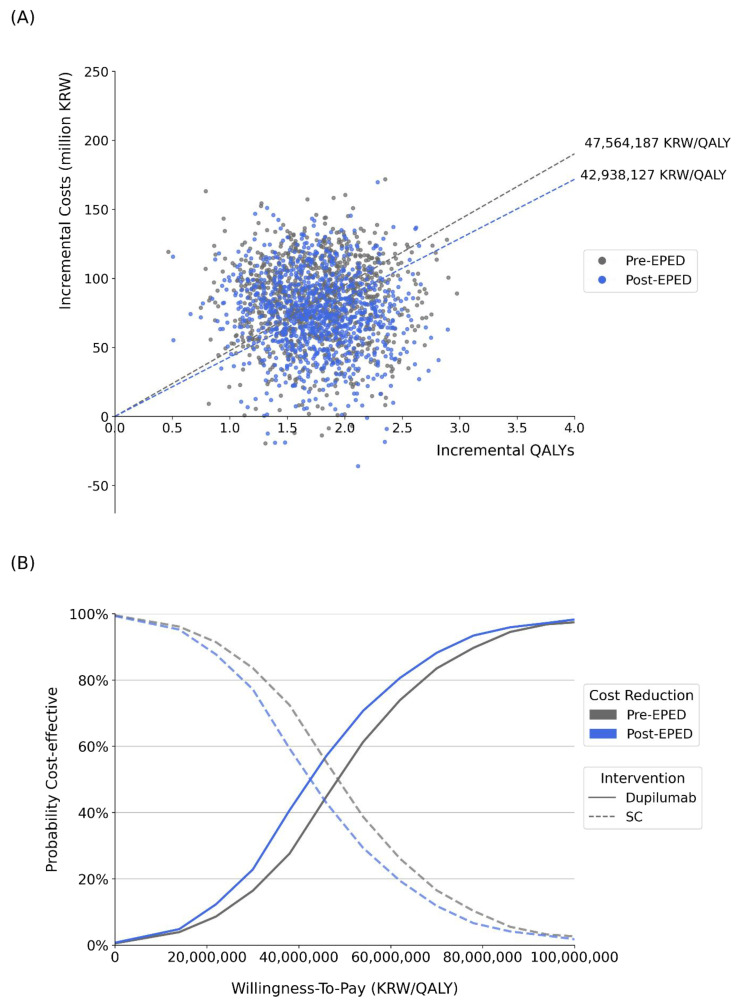
Probabilistic sensitivity analysis. (**A**) Dupilumab cost-effectiveness scatter plot by EPED adjustment. (**B**) Dupilumab acceptability curve.

Factoring in the EPED occurring after 10.3 years from 2021 led to a decrease of KRW 4.6 million/QALY (9.7% reduction) in the ICER (Table 2). In terms of acceptability, it rose from 30.7% to 45.3% at the 1 GDP per capita WTP threshold (Figure 3A,B). This improvement in cost-effectiveness was driven by cost savings of KRW 8.1 million (equivalent to USD 7093) in drug costs resulting from factoring in the EPED.

### 3.2. Impact of EPED on the Relationship of the ICER and Dupilumab’s Price

**Figure 4:**
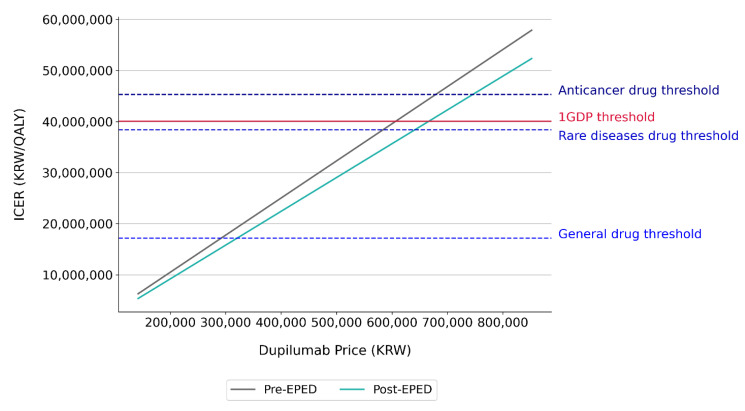
The unit price of dupilumab to meet WTP thresholds by EPED adjustment status.

The higher the annual cost of dupilumab, the more likely the treatment with dupilumab yields a higher ICER, resulting in an upward-sloping curve (Figure 4). The EPED adjustment then shifts the curve downward because it lowers the ICERs for each price of dupilumab. At the GDP threshold of KRW 40,052,159/QALY, the value-based price of dupilumab that would be considered cost-effective was KRW 606,656, which is 85.4% of the submitted price of KRW 710,000 without EPED. However, with the EPED adjustment, the value-based price increased to KRW 666,388, which was KRW 59,732 (8.4%) down from the submitted price (Table 3). When this difference is annualized, it amounts to approximately KRW 1,553,032 per person per year.

Given the WTP threshold for anticancer drugs, the drug needed a cost reduction of approximately KRW 31,000 per unit to achieve cost-effectiveness. However, the EPED adjustment made the drug cost-effective at its submitted price with a cushion of KRW 35,000. When different WTP thresholds were applied, the EPED adjustment returned the consistent improvement of 9.85% in value-based prices.

### 3.3. Impacts of Changes in EPED Configurations

Two factors affect EPED configurations: the time to EPED and the extent of price reduction at the EPED. Certainly, cost-effectiveness improves as the EPED occurs sooner. The ICER was KRW 39,085,632/QALY when EPED occurred at year 5 (Table 4), whereas the ICER was KRW 42,938,127/QALY when EPED occurred at 10.3 years, as computed from the remaining patent life of dupilumab in Korea.

EPED is implemented differently for a chemical as opposed to a biological like dupilumab. If the drug being evaluated for cost-effectiveness is a chemical (assuming dupilumab is a chemical drug), the ICER would have decreased by −14.9% to KRW 40,496,608 per QALY. This reduction is about 5% more than the biological dupilumab, approaching the proximity of the WTP threshold.

In terms of acceptability (the percentage at which the treatment is considered cost-effective at the ICER threshold of KRW 40,052,159 per QALY), the EPED adjustment increased acceptability to 45.3% from 30.7%. If dupilumab were a chemical, the EPED adjustment would have increased acceptability to 49.8%, a 4.5% increase compared to the biological dupilumab.

### 3.4. Other Factors Impacting the Cost-Effectiveness of Dupilumab

The cost-effectiveness of dupilumab would vary depending on how treatment benefit is defined, the duration of observation, and how time values are assigned to each cost and outcome occurring in the future. When the treatment benefit of dupilumab was less strictly defined, from EASI-75 to EASI-50, the ICER dropped by KRW 5,186,105/QALY from KRW 52,221,640/QALY, compared to the case without the EPED adjustment. However, the impact of the EPED adjustment was slightly smaller for the EASI-75 definition than for the EASI-50 (9.7% versus 9.9%) (Table 4).

**Figure 5:**
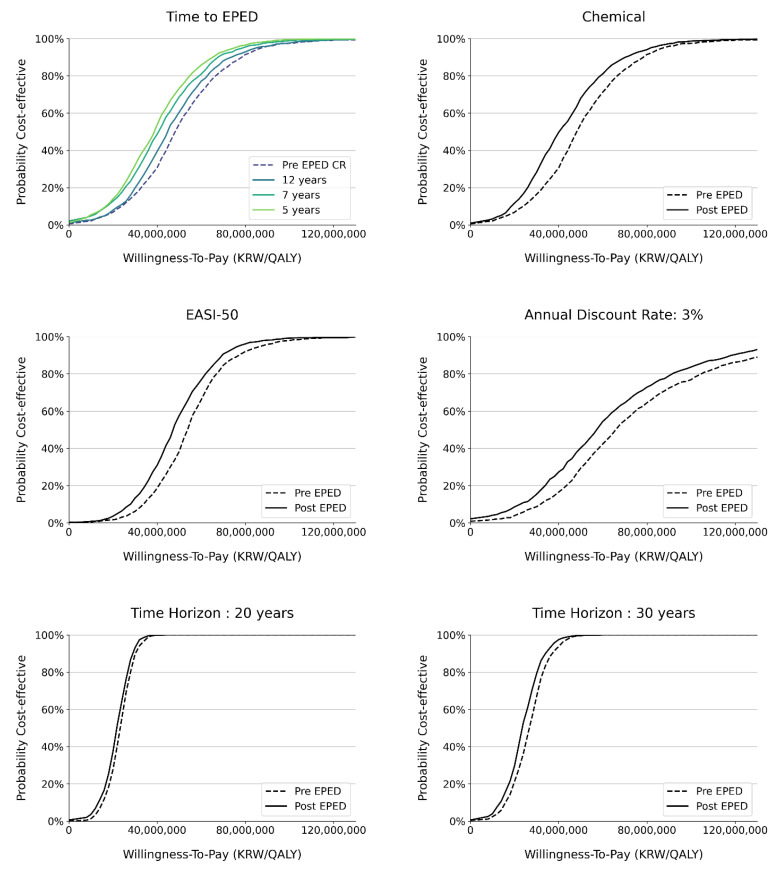
Changes in acceptability curve by scenarios.

The change in the time horizon also affected the ICER and the impact of the EPED adjustment. Extending the time horizon from 20 years to a lifetime raised the ICER from KRW 22,777,541/QALY to KRW 47,564,187/QALY. Nevertheless, the impact of the EPED adjustment became more significant for a longer time horizon (7.3% versus 9.7%). In terms of the impact on acceptability, the EPED adjustment made no improvement for the 20-year horizon but resulted in a 3.7% improvement for the 30-year horizon (Figure 5).

Regarding changes in the time value or discount rate, from 4.5% to 3%, the ICER increased from KRW 47,564,187/QALY to KRW 65,458,658/QALY. The EPED adjustment had a greater impact on reducing the ICER for lower time values; in other words, an 11.3% reduction for a 3% discount rate compared to a 9.7% reduction for a 4.5% discount rate.

### 3.5. One-Way Sensitivity Analysis

**Figure 6:**
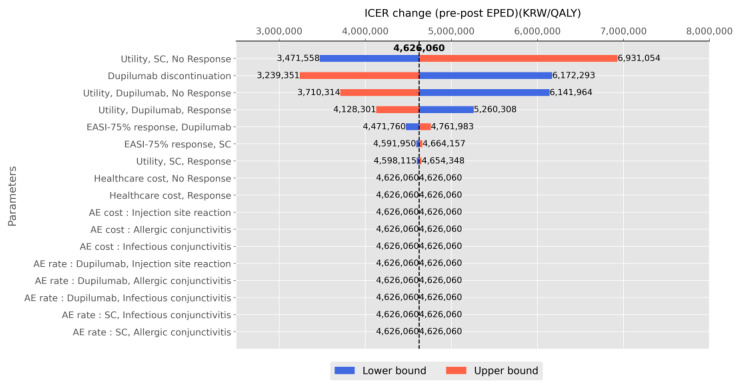
Tornado diagrams of one-way sensitivity analysis. The impact of model parameters on ICER change after EPED adjustment.

The impact of EPED on the ICER showed variation in response to uncertainty in model input parameters (Figure 6). The most significant increase in the ICER was observed for the upper 95% CI of the utility of the health state not responding to SC, followed by the lower bound of the utility of the health state for those who discontinued dupilumab. The changes in the ICER were considerable for the 95% CIs in the utility of those not responding to dupilumab and the utility of those who responded to dupilumab. Higher percentage of patients responding to dupilumab led to improved outcomes but also increased costs. However, the influences of healthcare costs, AE rates, and AE costs on the change in the ICER were negligible.

## 4. Discussion

The results of this study revealed that the implementation of post-entry cost reduction through the EPED policy improved the cost-effectiveness of dupilumab for the treatment of moderate to severe AD. The improvement in the ICER was KRW 4,626,060 less per QALY compared to the case without the adjustment, meaning that dupilumab’s acceptability increased by 14.6%.

The decrease in the ICER due to the EPED adjustment, however, was not as pronounced as in a UK study, where a substantial decrease in cost-effectiveness between 24% and 46% is reported. The UK study is based on an annual price reduction of 4%, while our study is based on a one-time price reduction of 30% occurring at the 10.3-year point from the time of economic evaluation. In the UK study, the drug cost begins to decrease as the cycle repeats, whereas in our study, it does not occur until more than ten cycles have passed. When the reduction in our study is converted to the UK equivalent annual rate, it amounts to a reduction of 1.27% per year for a 63-year time horizon.

The EPED adjustment returned the consistent percent improvement of 9.85% in value-based prices. Considering the WTP threshold for anticancer drugs, cost reduction of around KRW 31,000 (USD 27) per unit was required to be cost-effective. However, when the EPED was adjusted, the drug achieved cost-effectiveness at the submitted price. Also, the EPED adjustment returned the consistent percent improvement of 9.85% in value-based prices. These highlight the significance of implementing future costs, especially when a drug faces reimbursement rejection just above the WTP threshold.

Clearly, the extent to which the ICER of dupilumab fell depended on how the EPED was configured. EPED is characterized by two elements. The first element, the time to EPED, determines when the price reduction occurs. The second element, whether the drug is a chemical or biological drug, determines the percentage of the price reduction. As the time to EPED was shortened to 5 years compared to the base case of 10.3 years, the ICER of dupilumab substantially fell to KRW 39,398,450 per QALY, which was below the 1GDP threshold, resulting in a 57.9% increase in acceptability. On the other hand, if dupilumab were a chemical, EPED would reduce the future price by 49.8%, as opposed to 30.7% for the biological. As a result, the acceptability of the therapy increased by 19.1%.

In addition to the elements of EPED configuration, other factors such as the treatment response criterion, discount rate, and time horizon could influence the ICER. The ICER became worse for a more lenient criterion of treatment response, changing from EASI-75 to EASI-50. This may have occurred because the additional treatment response from dupilumab, relative to SC, was smaller for the EASI-50 criterion compared to the EASI-75 criterion [30]. In terms of the impact of the EPED adjustment, the relaxation in response criteria led to an improvement of 9.9% in the ICER; the acceptability improved by 12.9% with EASI-50 and 14.6% with EASI-75. As for the change in the time horizon, the ICER increased for a more extended time horizon. This indicates that the proportion of patients who would benefit from dupilumab shrinks due to discontinuation of the therapy. Nevertheless, the extent of the improvement in cost-effectiveness through EPED also increased as the implementation period was extended. This may have occurred because the number of years during which the EPED adjustment realizes its effect increases over time. Our study also found that the ICER increased when future values were discounted less strongly from 4.5% to 3%. This finding may have resulted from the declining value of dupilumab over time. Because dupilumab has the highest value at the initial stages, assigning weaker time values would result in less favorable economic evaluation outcomes. However, discounting future values less strongly led to a higher impact of the EPED adjustment on economic evaluation because the impact is realized in the future.

The sensitivity analysis revealed that incorporating uncertainty in the model input parameters yielded expected results. Specifically, the impact of EPED on ICER was found to be sensitive to uncertainties in the utilities of different health states for patients responding/not responding to SC and dupilumab, as well as response rates to SC and dupilumab treatment. However, it is noteworthy that healthcare costs, AE rates, and AE costs had a negligible impact on the ICER. This suggests that the model is robust in accounting for these parameters. Overall, while uncertainties surrounding health state utilities and patient response rates introduce variability in the cost-effectiveness results, the model appears robust in capturing the key determinants of cost-effectiveness for dupilumab treatment.

The time to EPED represents the period of market exclusivity remaining following the application to the HTA agency for drug coverage review. As market exclusivity expires sooner, the EPED adjustment would have a stronger impact on cost-effectiveness. In South Korea, the average effective patent life for approved drugs stands at 8.7 years, which is shorter than our case of 10.3 years for dupilumab [42]. Therefore, the majority would face generic drug entry within 10 years from the date of drug approval. The EPED adjustment thus holds the potential to enhance the cost-effectiveness of these drugs [42].

The economic evaluation of a new drug, adjusted for EPED, is particularly crucial when the drug faces rejection for NHI coverage due to its ICER slightly surpassing the WTP threshold. Even a minor improvement in the ICER through EPED adjustment could bring it within the acceptable range. This importance is amplified for chemical drugs, as the EPED-induced price reduction tends to be more substantial for them.

The study findings carry significant implications, particularly for evaluating the cost-effectiveness of high-cost anticancer drugs used in combination with best supportive care (BSC) versus BSC alone. Recently introduced anticancer drugs, such as targeted chemotherapy or immunotherapy, come with a hefty price tag, posing a considerable challenge for achieving cost-effectiveness. Incorporating entry-induced price reductions would undoubtedly bolster cost-effectiveness.

These study findings also apply to the pharmacoeconomic analysis of combination therapies involving backbone and add-on drugs. Compared to the backbone drug alone, combination therapy is more expensive but could become more affordable due to entry-induced price reduction of the add-on therapy. While the backbone therapy may also undergo entry-induced price reduction, potentially sooner than the add-on therapy, it does not alter the outcome as the reductions offset each other in both treatment options. Given the frequent introduction of such combinations, it is essential to recognize that EPED-induced future price reductions could substantially enhance the combination’s cost-effectiveness, especially with early generic entry for the add-on therapy.

The results of our study provide a simulated example of the impact of lifecycle drug pricing on cost-effectiveness evaluation. While many studies traditionally estimated future price reductions based on unreliable price data, our study used the EPED policy that mandates a categorical price reduction for drugs with identical active ingredients and formulations [5,6,9,43]. Our approach thus eliminates the uncertainty associated with the future drug price estimation and thus serves as a potentially viable case for drug lifecycle pricing.

While our study provides a comprehensive analysis, it is important to acknowledge certain limitations. First, our analysis was specifically focused on dupilumab for atopic dermatitis, and the findings may not be directly applicable to other cases involving one-time or short-term treatments. For instance, treatments such as antibiotics for acute infections, pain relief medications for temporary conditions, or vaccinations for immunization typically do not necessitate prolonged or continuous use. As a result, the cost-effectiveness dynamics of these interventions may differ from that of chronic therapies like dupilumab.

Furthermore, additional price reductions could occur in the future after the EPED-induced price reduction, which were not included in this study. However, such additional price reductions rarely occur in Korea because the industry fears they would lower drug reimbursement costs [18]. Moreover, the inclusion of the additional price reductions would likely yield a favorable impact on dupilumab because the reductions only apply to dupilumab.

We assumed that patients on dupilumab do not switch to generic drugs despite their entry. This assumption is reasonable because patients have no incentive to switch when both drugs are priced equally, as mandated by the EPED policy for biologicals like dupilumab, which imposes a categorical price reduction of 30% upon entry. However, for chemical drugs, there is differential pricing between the original drug and the first generic version for the first year (70% for the original and 59.5% for the first generic). Therefore, it is possible that patients may switch to the cheaper generic version during this period. However, the impact of such switching would likely be insignificant because both drugs are subject to a categorical reduction of 47% after the first year [15].

It is worth considering that patients may switch to next-line therapies or competing drug therapies before the EPED occurs, which could nullify the impact of EPED. Alternatively, switches may occur after EPED, potentially biasing the EPED adjustment’s impact. However, for the purposes of this study, we assumed that such switches do not happen.

This study estimated the value-based price of dupilumab based on the assumption that the price used in this study was the one submitted to the HIRA. However, the actual price is unknown. It is possible that the actual price could have been lower, particularly considering that the HIRA entered a risk-sharing agreement for dupilumab [44]. A lower price would likely result in more favorable ICERs and acceptability outcomes compared to our study results, although it would also reduce the impact of EPED.

Our case represents a specific instance of this scenario, where the future price reduction is pending for the new drug while it has already occurred for the comparator. This scenario is applicable when the comparator is best supportive care (BSC) or backbone therapy included in combination therapy. However, it does not apply when the future price reductions would affect both the new drug and the comparator simultaneously. In such cases, the EPED adjustment may have a negligible impact, as both alternatives undergo price reductions. Moreover, it could even negatively affect the cost-effectiveness of the new drug if the brand comparator undergoes price reduction sooner than the new drug.

## 5. Conclusions

In conclusion, incorporating future cost reductions resulting from generic entry through the EPED policy significantly improved the cost-effectiveness of dupilumab, and increased the probability of it being considered cost-effective at the WTP threshold. These findings lend support to the implementation of “drug life-cycle pricing” to ensure balanced cost-effectiveness study to innovative medications. Given that EPED is mandatory and enforced by the HIRA, it is recommended to reflect the EPED-induced price reductions in the HIRA guidelines for economic evaluations. This inclusion would facilitate a fair and balanced economic assessment between payers and industries, ultimately enhancing patient access to innovative medications.

## Figures and Tables

**Table 1:** Model Input.

Parameters	Mean	Distribution	References
EASI-75 response			
Dupilumab group	47.7%	Beta (alpha = 218, beta = 239)	[30]
SC group	13.3%	Beta (alpha = 61, beta = 399)	[30]
Annual discontinuation probability of dupilumab	6.3%	Beta (alpha = 24, beta = 357)	[28,29]
Relapse after 16-week of emollient	36.7%	Beta (alpha = 40, beta = 69)	[32]
Adverse event rate			
Dupilumab			
Injection site reaction	11.0%	Beta (alpha = 51, beta = 414)	[30]
Allergic conjunctivitis	3.0%	Beta (alpha = 14, beta = 451)	[30]
Infectious conjunctivitis	4.3%	Beta (alpha = 20, beta = 445)	[30]
SC			
Injection site reaction	-	-	[28,29]
Allergic conjunctivitis	0.9%	Beta (alpha = 4, beta = 452)	[30]
Infectious conjunctivitis	0.7%	Beta (alpha = 3, beta = 453)	[30]
Cost (KRW)			
a 300-mg injection of dupilumab			
Pre-entry	710,000	-	[34]
Post-entry	497,000	-	-
Healthcare cost per cycle			
No response (SE)	1,058,567	Gamma (SE = 395,690)	[35]
Response (SE)	522,041	Gamma (SE = 135,946)	[35]
Adverse event annual cost (KRW)			
Injection site reaction	39,512	Gamma (SE assumed to be 10% of mean)	[36]
Allergic conjunctivitis	25,129	Gamma (SE assumed to be 10% of mean)	[36]
Infectious conjunctivitis	22,524	Gamma (SE assumed to be 10% of mean)	[36]
Utility			
Dupilumab			
No response (SE)	0.63	Gamma (SE = 0.015)	[37]
Response (SE)	0.89	Gamma (SE = 0.024)	[37]
SC			
No response (SE)	0.61	Gamma (SE = 0.016)	[37]
Response (SE)	0.86	Gamma (SE = 0.046)	[37]

**Table 2:** Economic evaluation of dupilumab plus SC versus SC by pre- and post-EPED.

Outcome ^1^	Pre-EPED		Post-EPED		Difference
	Dupilumab	SC	Dupilumab	SC	
Drug Cost	90,579,395	-	82,460,271	-	
Other Healthcare Cost	54,179,490	61,279,752	54,179,490	61,279,752	
Total Cost	144,758,885	61,279,752	136,639,761	61,279,752	
Total QALY	13.33	11.58	13.33	11.58	
Incremental Cost	83,479,133		75,360,009		−8,119,123
Incremental QALY	1.76		1.76		
Incremental Cost-Effective Ratio	47,564,187		42,938,127		−4,626,060 (−9.7%)
Acceptability Change ^2^ (%)	30.7%		45.3%		+14.6′%

^1^ All costs listed in the table are in KRW. ^2^ Evaluated at the 1GDP threshold of KRW 40,052,159 per QALY.

**Table 3:** Value-based price based on different WTP thresholds.

	Value Based Price (KRW)		Difference		
WTP Thresholds	Pre-EPED	Post-EPED	per Unit (KRW)	% Change	per Year (KRW)
1GDP-based	606,656	666,388	59,732	+9.85%	1,553,032
Anticancer-based	679,126	745,994	66,867	+9.85%	1,738,555
Rare disease-based	583,927	641,421	57,494	+9.85%	1,494,846
General disease-based	291,864	320,601	28,737	+9.85%	747,169

**Table 4:** Scenario analysis results.

Variables	Pre-EPED ICER (KRW/QALY)	Post-EPED ICER (KRW/QALY)	ICER Change (KRW/QALY) (%)	Acceptability Change (%)
EPED Configuration				
Time to EPED				
5 years	47,564,187	39,085,632	−8,478,555 (−17.8%)	23.1%
7 years	47,564,187	40,801,064	−6,763,123 (−14.2%)	18.0%
12 years	47,564,187	43,744,542	−3,819,645 (−8.0%)	8.8%
Type of EPED				
Chemical-based	47,564,187	40,496,608	−7,067,579 (−14.9%)	19.1%
Scenario Analysis				
Time Horizon				
30 years	26,058,505	23,690,242	−2,368,264 (−9.1%)	3.7%
20 years	22,777,541	21,117,578	−1,659,963 (−7.3%)	0%
Response				
EASI-50	52,221,640	47,035,535	−5,186,105 (−9.9%)	12.9%
Discount Rate				
3%	65,458,658	58,078,211	−7,380,447 (−11.3%)	10.8%

## Data Availability

All data generated or analyzed during this study are included in this published article and its Supplementary Materials files.

## Supplementary Materials

The following supporting information can be downloaded at: https://www.mdpi.com/article/10.3390/healthcare12090938/s1, Table S1: CHEERS 2022 Checklist; Table S2: Health Insurance Claims and Total Healthcare Costs for Adverse Events in South Korea in 2021; Information S1. Future price reduction scenario based on the EPED policy.
