# Supplementary material for: Impact of Incorporating Future Mandatory Price Reductions with Generic Drug Entry on the Cost-Effectiveness of New Drugs: A Policy Simulation Study of Dupilumab in Atopic Dermatitis Treatment
Source: Healthcare (Basel). 2024 May 2;12(9):938. doi: 10.3390/healthcare12090938 (PMC11083512; doi:10.3390/healthcare12090938)
Supplement: Supplementary file 1 [file healthcare-12-00938-s001.zip › healthcare-2932088-supplementary.pdf]

Supplementary Materials

Title : Impact of Incorporating Future Mandatory Price Reductions with Generic Drug Entry on the Cost-Effectiveness of New Drugs: A Policy Simulation Study of Dupilumab in Atopic Dermatitis Treatment

Journal: Healthcare

Supplementary Table S1. CHEERS 2022 Checklist

Topic	No.	Item	Location where item is reported
**Title**			
	1	Identify the study as an economic evaluation and specify the interventions being compared.	Title
**Abstract**			
	2	Provide a structured summary that highlights context, key methods, results, and alternative analyses.	Abstract
**Introduction**			
**Background and objectives**	3	Give the context for the study, the study question, and its practical relevance for decision making in policy or practice.	Introduction
**Materials and Methods**			
**Health economic analysis plan**	4	Indicate whether a health economic analysis plan was developed and where available.	Materials and Methods; Model Structure
**Study population**	5	Describe characteristics of the study population (such as age range, demographics, socioeconomic, or clinical characteristics).	Materials and Methods; Patients
**Setting and location**	6	Provide relevant contextual information that may influence findings.	Materials and Methods; Study Design
**Comparators**	7	Describe the interventions or strategies being compared and why chosen.	Materials and Methods; Study Design
**Perspective**	8	State the perspective(s) adopted by the study and why chosen.	Materials and Methods; Study Design
**Time horizon**	9	State the time horizon for the study and why appropriate.	Materials and Methods; Model Structure
**Discount rate**	10	Report the discount rate(s) and reason chosen.	Materials and Methods; Model Structure
**Selection of outcomes**	11	Describe what outcomes were used as the measure(s) of benefit(s) and harm(s).	Materials and Methods; Model Structure
**Measurement of outcomes**	12	Describe how outcomes used to capture benefit(s) and harm(s) were measured.	Materials and Methods; Transition Probabilities, Adverse Events, QALY Estimation

Topic	No.	Item	Location where item is reported
**Valuation of outcomes**	13	Describe the population and methods used to measure and value outcomes.	Materials and Methods; Patients
**Measurement and valuation of resources and costs**	14	Describe how costs were valued.	Materials and Methods; Cost Estimation
**Currency, price date, and conversion**	15	Report the dates of the estimated resource quantities and unit costs, plus the currency and year of conversion.	Materials and Methods; Cost Estimation
**Rationale and description of model**	16	If modelling is used, describe in detail and why used. Report if the model is publicly available and where it can be accessed.	Materials and Methods; Model Structure
**Analytics and assumptions**	17	Describe any methods for analysing or statistically transforming data, any extrapolation methods, and approaches for validating any model used.	Materials and Methods; Analysis
**Characterising heterogeneity**	18	Describe any methods used for estimating how the results of the study vary for subgroups.	Not applicable
**Characterising distributional effects**	19	Describe how impacts are distributed across different individuals or adjustments made to reflect priority populations.	Not reported
**Characterising uncertainty**	20	Describe methods to characterise any sources of uncertainty in the analysis.	Materials and Methods; Analysis
**Approach to engagement with patients and others affected by the study**	21	Describe any approaches to engage patients or service recipients, the general public, communities, or stakeholders (such as clinicians or payers) in the design of the study.	Not reported
**Results**			
**Study parameters**	22	Report all analytic inputs (such as values, ranges, references) including uncertainty or distributional assumptions.	Table 1, Supplementary table 1
**Summary of main results**	23	Report the mean values for the main categories of costs and outcomes of interest and summarise them in the most appropriate overall measure.	Table 2, Figure 3
**Effect of uncertainty**	24	Describe how uncertainty about analytic judgments, inputs, or projections affect findings. Report the effect of choice of discount rate and time horizon, if applicable.	Table 3,4 Figure 4,5

Topic	No.	Item	Location where item is reported
**Effect of engagement with patients and others affected by the study**	25	Report on any difference patient/service recipient, general public, community, or stakeholder involvement made to the approach or findings of the study	Not reported
**Discussion**			
**Study findings, limitations, generalisability, and current knowledge**	26	Report key findings, limitations, ethical or equity considerations not captured, and how these could affect patients, policy, or practice.	Discussion, Limitation
**Other relevant information**			
**Source of funding**	27	Describe how the study was funded and any role of the funder in the identification, design, conduct, and reporting of the analysis	Title page(s)
**Conflicts of interest**	28	Report authors conflicts of interest according to journal or International Committee of Medical Journal Editors requirements.	Title page(s)

Supplementary Table S2. Health Insurance Claims and Total Healthcare Costs for Adverse Events in South Korea in 2021

	Number of health insurance claims	Total Healthcare Cost (KRW 1,000)
**Injection site reaction**		
피부 및 피하조직의 국소적 부기, 종괴 및 덩이 (R22)<br>Localized swelling, masses, and lumps in the skin and subcutaneous tissue	211	8,337
**Allergic conjunctivitis**		
급성 아토피결막염 (H101)<br>Acute Atopic Conjunctivitis	2,821,890	70,910,574
**Infectious conjunctivitis**		
아데노바이러스에 의한 각막결막염(B300)<br>Adenoviral keratoconjunctivitis	90,691	22,296
아데노바이러스에 의한 결막염(B301)<br>Conjunctivitis caused by adenovirus	11,975	23,052
기타 바이러스결막염(B308)<br>Other viral conjunctivitis	11,880	20,660
상세불명의 바이러스결막염(B309)<br>Unspecified viral conjunctivitis	15,390	20,203
달리 분류된 기타 감염성 및 기생충 질환에서의 각막염 및 각막결막염(H131)<br>Keratitis and keratoconjunctivitis in other infectious and parasitic diseases not elsewhere classified	25,874	25,400
급성 유행성 출혈성 결막염(엔테로바이러스)(B303)<br>Acute epidemic hemorrhagic conjunctivitis (Enterovirus)	1,464	21,077

Supplementary Information S1. Future price reduction scenario based on the EPED policy

In this study, we reexamine the mathematical framework introduced by Shih et al.[i] and Guertin et al.[ii] Let,

T = end of the model's time horizon

$C_j(t)$ = mean total cost of the treatment j at time t

$ND_j(t)$ = mean nondrug costs of the treatment j at time t

$P_j(t)$ = price of the treatment j at time t

$Q_j(t)$ = mean total quantity of the treatment j consumed at time t

$E_j(t)$ = mean effectiveness of the treatment j at time t

r = discount rate

j = 1 if new drug or 0 if the comparator drug or supportive care

ΔC = incremental cost when ignoring the future price reduction

ΔC' = incremental cost when considering the future price reduction

ΔE = incremental effectiveness when ignoring the future price reduction

ΔE' = incremental effectiveness when considering the future price reduction

ICER = ICER when ignoring the future price reduction

ICER' = ICER when considering the future price reduction

When ignoring the EPED adjustment after generic entry, the price of the originator will remain constant throughout the observation timeline ($P_1(t) = P_1$). This leads to the estimation of the incremental cost, incremental effectiveness, and incremental cost-effectiveness ratio (ICER) as follows:

$$\Delta \mathrm{C} = \sum_{t=0}^{T} \frac{P_1(t) \cdot Q_1(t)}{(1+r)^t} - \sum_{t=0}^{T} \frac{P_0(t) \cdot Q_0(t)}{(1+r)^t} + \sum_{t=0}^{T} \frac{ND_1(t) - ND_0(t)}{(1+r)^t}$$

$$\Delta \mathrm{E} = \sum_{t=0}^{T} \frac{E_1(t) - E_0(t)}{(1+r)^t}$$

$$\mathrm{ICER} = \frac{\Delta \mathrm{C}}{\Delta \mathrm{E}}$$

With the assumption of EPED adjustment, it is hypothesized that the price of the new drug would be subjected to a price reduction within the observation period ($T^* < T$), while the comparator drug, which already experienced price reduction, would not be affected by such change ($P_0(t) = P_0$).

Then new drug price (P_1) can be defined as:

$$Biological\ drugs:\ P_1(t) = \begin{cases} P_1 & (t < T^*) \\ 0.7 * P_1 & (t \geq T^*) \end{cases}$$

$$Chemical\ drugs:\ P_1(t) = \begin{cases} P_1 & (t < T^*) \\ 0.7 * P_1 & (T^* \leq t < T^* + 12\ month) \\ 0.5355 * P_1 & (t \geq T^* + 12\ months) \end{cases}$$

Subsequently, the incremental cost, incremental effectiveness and ICER will be recalculated based on the EPED adjusted price of the new drug and the comparator drug. Incremental cost reflecting cost reduction (ΔC') will vary from ΔC while the effectiveness remains the same. (ΔE = ΔE'). For example, ΔC' for the biologic drugs can be defines as:

$$\Delta C' = \sum_{t=0}^{T*-1} \frac{P_1(t) \cdot Q_1(t)}{(1+r)^t} + \sum_{t=T*}^{T} \frac{0.7P_1(t) \cdot Q_1(t)}{(1+r)^t} - \sum_{t=0}^{T} \frac{P_0(t) \cdot Q_0(t)}{(1+r)^t}$$
$$+ \sum_{t=0}^{T} \frac{ND_1(t) - ND_0(t)}{(1+r)^t}$$

Since $P_{1P} \geq 0$ and $Q_1(t) \geq 0$, the difference between ΔC and ΔC' is always nonnegative, which can be presented as:

$$\Delta C - \Delta C' = \sum_{t=T*}^{T} \frac{0.3P_1 \cdot Q_1(t)}{(1+r)^t} \geq 0$$

In conclusion, pre-EPED ICER is larger than or equal to post-EPED ICER because of the following formula:

$$\mathrm{ICER} = \frac{\Delta \mathrm{C}}{\Delta \mathrm{E}} \geq \frac{\Delta \mathrm{C}'}{\Delta \mathrm{E}'} = \mathrm{ICER}'$$

---

[i] Shih, Y.-C. T., Han, S. & Cantor, S. B. Impact of Generic Drug Entry on Cost-Effectiveness Analysis. *Med Decis Making* **25**, 71–80 (2005).

[ii] Guertin, J. R., Mitchell, D., Ali, F. & LeLorier, J. Bias within economic evaluations – the impact of considering the future entry of lower-cost generics on currently estimated incremental cost-effectiveness ratios of a new drug. *Clinicoecon Outcomes Res* **7**, 497–503 (2015).
